# Skeletal structure of asymmetric mandibular prognathism and retrognathism

**DOI:** 10.1186/s40902-023-00393-7

**Published:** 2023-08-09

**Authors:** Tong Xi, Shankeeth Vinayahalingam, Stefaan Bergé, Thomas Maal, Tae-Geon Kwon

**Affiliations:** 1 Department of Oral and Maxillofacial Surgery, Radboud University Nijmegen Medical Centre, Geert Grooteplein 10, 6525 GA Nijmegen, The Netherlands; 2 3D Imaging Lab, Radboud University Nijmegen Medical Centre, Geert Grooteplein 10, 6525 GA Nijmegen, The Netherlands; 3 Department of Oral and Maxillofacial Surgery, School of Dentistry, Kyungpook National University, 2177 Dalgubeol-Daero, Jung-Gu, Daegu, 41940 Republic of Korea

**Keywords:** Asymmetry, Condyle, Ramus, Mandibular prognathism, Retrognathism

## Abstract

**Background:**

This study aimed to compare the skeletal structures between mandibular prognathism and retrognathism among patients with facial asymmetry.

**Results:**

Patients who had mandibular asymmetry with retrognathism (Group A) in The Netherlands were compared with those with deviated mandibular prognathism (Group B) in Korea. All the data were obtained from 3D-reformatted cone-beam computed tomography images from each institute. The right and left condylar heads were located more posteriorly, inferiorly, and medially in Group B than in Group A. The deviated side of Group A and the contralateral side of Group B showed similar condylar width and height, ramus-proper height, and ramus height. Interestingly, there were no inter-group differences in the ramus-proper heights. Asymmetric mandibular body length was the most significantly correlated with chin asymmetry in retrognathic asymmetry patients whereas asymmetric elongation of condylar process was the most important factor for chin asymmetry in deviated mandibular prognathism.

**Conclusion:**

Considering the 3D positional difference of gonion and large individual variations of frontal ramal inclination, significant structural deformation in deviated mandibular prognathism need to be considered in asymmetric prognathism patients. Therefore, Individually planned surgical procedures that also correct the malpositioning of the mandibular ramus are recommended especially in patients with asymmetric prognathism.

## Background

Similar to other facial deformities, mandibular asymmetry is related to congenital or acquired etiological backgrounds [1, 2]. In the absence of a congenital syndrome, or trauma or disease after birth, mandibular asymmetry can occur as a result of unilateral over- or under-growth of the mandible due to unknown causes. Mandibular asymmetry with prognathism is not only frequently accompanied by changes in symphyseal chin morphology, but also shows condylar, ramal, and body deformation [3–6]. Facial asymmetry with retrognathism may be associated with unilateral temporomandibular joint (TMJ) disorders and frequently exhibits condylar height shortening on the ipsilateral side of chin deviation [1, 7, 8].

It has been reported that the chin deviation in skeletal class III patients increases as age increases, whereas no age-dependent increases in chin deviation have been reported in skeletal class II patients. This may be attributed to the fact that class III patients are more likely to be exposed to longer periods of postnatal or environmental influences [9, 10]. Therefore, it would be reasonable to conclude that the pattern of facial asymmetry in mandibular retrognathism would have characteristics different from those of prognathism.

However, the differences in structural asymmetry between the retrognathic and prognathic mandibles have not been properly elucidated. A three-dimensional (3D) analysis using computed tomography (CT)-reformatted images is critical in analyzing the complex pattern of asymmetry. However, a comprehensive examination of the 3D facial skeleton using cone-beam CT (CBCT) has not been performed frequently in previous studies. This might be attributed to the insufficient number of 3D studies investigating mandibular asymmetry in retrognathic patients.

Till date, only few comparative studies between mandibular prognathism and retrognathism in patients with facial asymmetry have been performed using 3D CT images [11]. The prevalence of class III occlusion is higher in east Asian populations [12]. but relatively low in western countries [13, 14]. This study tried to take an advantage of the popular type of patients in Asia and Europe.

The purpose of this study was to investigate whether there are any differences in the mandibular structures of patients with deviated mandibular prognathism and retrognathism by using 3D measurements. We also evaluated the possible correlation between mandibular deviation and 3D anatomical measurements of asymmetric mandibles in both groups.

## Methods

### Study subjects

A total of 74 patients with facial asymmetry who had undergone orthognathic surgery from January 2015 to June 2016 at the authors’ affiliated hospitals were recruited for the study. Facial asymmetry was defined as chin deviation (Me) greater than 3 mm with respect to the facial midsagittal reference plane [15] over than 17 years of age. To enroll patients with mandibular asymmetry with prognathism (ANB < 0°) for Group A, mandibular hyperplasia, class III occlusion were included at Kyungpook National University Hospital (KNUH), Daegu, Korea. For group B, mandibular asymmetry with retrognathism (ANB > 3°) were enrolled. patients, mandibular hypoplasia, class II occlusion of were included at Nijmegen Medical Centre (NMC), Radboud University, Nijmegen, The Netherlands.

Patients who had syndromes, cleft lips and/or palates, hemifacial microsomia, congenital muscular torticollis, or previous history of facial trauma or infection, or had undergone TMJ surgery were excluded. The study was approved by the ethics committee of the institutional review board at KNUH in Korea (KNUH_2016-08–017) and by the regional medical ethics review board in The Netherlands (CMO Arnhem-Nijmegen, 181/2005).

### 3D CT image analysis

Group A patients underwent CT examination using a CBCT scanner (CB Mercuray, Hitachi Medico, Tokyo, Japan) at KNUH, in Korea: 19-cm field of view, 120-kVp tube voltage, 15-mA tube current and 0.4 mm voxel size. Group B patients were imaged with CT using a CBCT scanner (i-CAT, 3D Imaging System, Imaging Sciences, International Inc., Hatfield, PA, USA) at NMC in The Netherlands: 22-cm field of view, 120-kVp tube voltage, 8-mA tube current and 0.4 mm voxel size. After scanning, the CBCT data were exported in digital imaging and communications in medicine (DICOM) file format and reconstructed into a 3D image using the Maxilim® software (Medicim NV, Mechelen, Belgium).

**Fig. 1:**
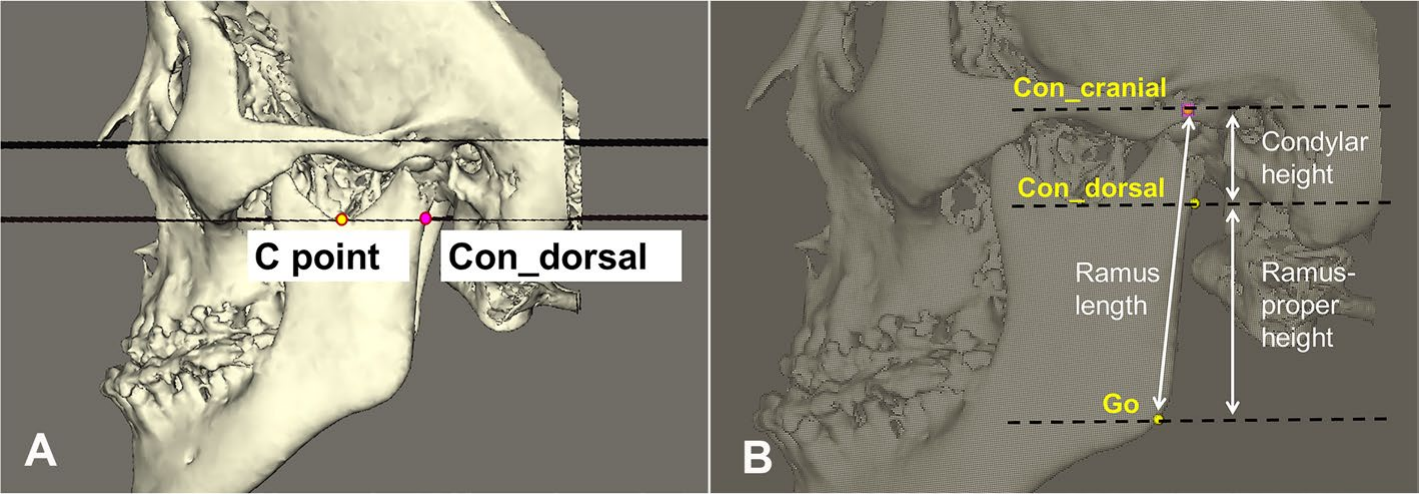
Reference points and measurements of the condyle region. **A** C-plane: the plane that runs through C_point and con_dorsal and parallel to the Frankfurt plane. **B** Condylar height, the height between landmarks Con_cranial and Con_dorsal (i.e., distance between the two landmarks along the y-axis of the reference frame); Ramus-proper height, the height between landmarks Con_dorsal and Go; Ramus length, the distance from Con_cranial to Go

**Fig. 2:**
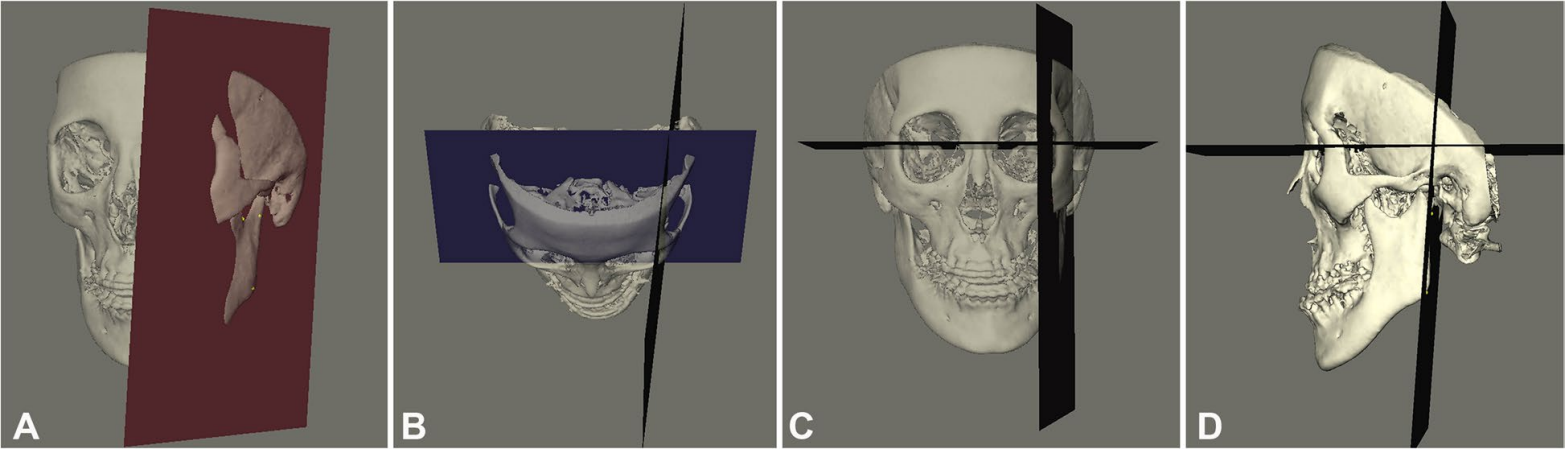
Angular measurements for the study. **A** Ramus sagittal plane (Ramus_sag_plane): a plane through landmarks C_point, Con_dorsal, and Go. **B** Axial ramal rotation (yaw): The 3D angle between plane Ramus_sag_plane and CRP. **C** Frontal ramal inclination (roll): The 3D angle between plane Ramus_sag_plane and HRP. **D** Sagittal ramal inclination (pitch): The angle between the projection of the lines posterior ramus (Con_dorsal to Go) on medial plane and S–N from the lateral view

The 3D-rendered head models were reoriented to reference planes as reported in previous studies [16]. The definitions of reference plane and measurements including condyle and ramus sagittal plane relative to the reference planes are defined in Table 1 and Fig. 1, similar to previous reports [17, 18]. A ramus sagittal plane was defined as a plane that passes through the most dorsal point of the condyle (Con_dorsal), the sigmoid notch (C_point) and gonion (Go). The condylar height was defined as the vertical height between the most cranial point of the condyle (Con_cranial) and the Con_dorsal. Ramus-proper height was defined as the height from Con_dorsal to Go, which excludes the vertical condylar height (Go_cranial to Con_dorsal) and represents the ramal growth lower than the subcondylar area (Fig. 2). The distance between Con_cranial to Go was defined as the ramus length, which is similar to that of other studies [5, 6, 11, 19, 20]. The “deviated side” was defined as the side of the mandibular midline shift from the MSP, whereas “contralateral side” referred the side opposite the chin deviation [19, 20].

**Table 1:** Definitions of 3D landmarks, planes, and measurements

Annotation	Definition
Landmarks	
Na	The midpoint of the frontonasal suture
S	The center of the hypophyseal fossa (sella turcica)
Or	The orbitale. Bilateral landmark
Pog	The hard tissue pogonion
Por	The porion. Bilateral landmark
Por_center	The geometric mean of left and right porion. Bilateral landmark
A-point	The hard tissue A-point
B-point	The hard tissue B-point
C_point	The most caudal point of the sigmoid notch. Bilateral landmark
Con_ant	The most anterior point of the condyle. Bilateral landmark
Con_post	The most posterior point of the condyle. Bilateral landmark
Con_AP_center	The transverse geometric center of the condyle (between Con_ant and Con_post). Bilateral landmark
Con_cranial	The most cranial point of the condyle. Bilateral landmark
Con_dorsal	The dorsal point of the condyle at the intersection with C-plane. Bilateral landmark
Con_lat	The most lateral point of the condyle. Bilateral landmark
Con_med	The most medial point of the condyle. Bilateral landmark
Cor	The tip of the coronoid process. Bilateral landmark
Me	The most inferior midpoint of the chin at the mandibular symphysis. Hard tissue menton
Go	The most posterior and inferior point of the mandibular angle. Bilateral landmark
Reference planes	
FH Plane	A plane through landmarks right and left Or and Por_Center
Horizontal reference plane(HRP)	The Horizontal (x) 3-D Cephalometric Reference Plane is automatically computed as a plane 6 degrees below the Anterior Cranial Base (S-Na) plane, through S
Midsagittal plane (MRP)	The Median 3D Cephalometric Reference Plane is computed as a plane through S and Na and perpendicular to the HRP
Coronal reference plane (CRP)	The Vertical 3D Cephalometric Reference Plane is computed as a plane through S and perpendicular to the HRP and MRP
MP	Mandibular plane. A plane through landmarks right Go, left Goand Me. Bilateral plane
C-plane	A plane through landmark C_pointand parallel to FH plane. Bilateral plane
Condyle_sag_plane	Condyle sagittal plane, a plane through Con_center, Con_dorsal and C_point. Bilateral plane
Ramus_sag_plane	Ramus sagittal plane, a plane through landmarks C_point, Con_dorsal and Go. Bilateral plane
Linear measurements (mm)	
Me deviation	The distance from landmark Me to MRP
Chin height	The height between landmarks B-point and Me (i.e. distance between the two landmarks along the y-axis of the reference frame)
Condylar width	The distance between the most medial and lateral point of the condyle (Con_med to Con_lat). Bilateral measurement
Condylar height	The height between landmarks Con_cranial and Con_dorsal (i.e., distance between the two landmarks along the y-axis of the reference frame). Bilateral landmark
Ramus-proper height	The height between landmarks Con_dorsal and Go (i.e., distance between the twolandmarks along the *y*-axis of the reference frame). Bilateral landmark
Ramus length	The distance from Con_cranial to Go. Bilateral measurement
Body length	The length of left mandibular body (Go to Me). Bilateral measurement
Con_AP	The anterio-posterior depth between landmark Con_sag_mean to N vertical from lateral view. Bilateral measurement
Con_vert	The vertical distance between landmark Con_cranial to N along the *x*-axis of the reference frame (The vertical distance between condyle_cranial and axial plane at N). Bilateral measurement
Con_transv	The distance from landmark Con_cranial to MRP. Bilateral measurement
Go_AP	The anterio-posterior depth between landmarks Go and N vertical (i.e., distance between the two landmarks along the *x*-axis of the reference frame). Bilateral measurement
Go_vert	The height between landmarks N and Go (i.e., distance between the two landmarks along the y-axis of the reference frame). Bilateral landmark
Go_transv	The distance from landmark Go_to plane MRP
Angular measurements (°)	
SNA	The angle between the projection of the landmarks S–N-A-point on MRP
SNPog	The angle between the projection of the landmarks S–N-Pog on MRP
MPA	Mandibular plane angle, the angle between the line S–N and the MP
Axial condyle rotation (yaw)	The 3D angle between plane Condyle_sag_plane and CRP. Rotation of condylar-sagittal plane relative to CRP from bird’s eye view. Bilateral measurement
Frontal condyle inclination(roll)	The 3D angle between plane Condyle_sag_plane and HRP. Coronal rotation of left condylar-ramus sagittal plane with regard to HRP from frontal view. Bilateral measurement
Axial ramal rotation (yaw)	The 3D angle between plane Ramus_sag_plane and CRP. Bilateral measurement
Frontal ramal inclination (roll)	The 3D angle between plane Ramus_sag_plane and HRP. Bilateral measurement
Sagittal ramal inclination (pitch)	The angle between the projection of the lines posterior ramus (Con_dorsal to Go) on medial plane and S–N from the lateral view. Bilateral measurement
Gonial angle	The angle between the projection of the lines Con_dorsal-Go and Go-Me on MRP. Bilateral measurement

### Data analysis and statistics

The data were statistically analyzed using SPSS software (version 12.0; SPSS Inc., Chicago, IL, USA). To prevent inter-observer error, all procedures were performed by one investigator (T. X.). The bilateral difference within groups was analyzed using the paired *t*-test for normally distributed data and Wilcoxon-signed rank test for not normally distributed data. The Inter-group differences were compared with independent *t* test for normally distributed data and Mann–Whitney *U* test for non-normally distributed data. The relationship between the measurements were evaluated using the Pearson correlation analysis. The level of significance was set at 0.05 for all statistical analyses.

To evaluate the reproducibility of the measurements, all landmarks and measurements were examined twice at an interval of one week by the same investigator for the first 10 patients. Intra-class correlations and mean differences between the two sets of measurements were calculated to determine the reliability of data. The interclass correlation ranged from 0.875 to 0.999, and showed that the difference between the repeated measurements were not statistically significant (all *p* > 0.05).

## Results

### Differences in demographic characteristics of both groups

The demographic data of the patients including the amount of transverse Me deviation in both groups are listed in Table 2. There was a significant difference in ANB between the two groups. However, there were no statistically significant differences in Me deviation, age, gender distribution, maxillary horizontal position, mandibular plane angle, and chin height.

**Table 2:** Demographic and skeletal characteristics of the two groups

Characteristics	Group A (asymmetry with prognathism)	Group B (asymmetry with retrognathism)	*p*
*N*	38	36	
Female (%, *n*)	20 (52.7%)	12 (33.3%)	0.094
Age (years)	22.8 ± 2.5 (19 ~ 30)	25.0 ± 8.3 (17 ~ 49)	0.144
SNA (°)	80.7 ± 3.4 (74.0 ~ 89.1)	81.7 ± 4.3 (72.3 ~ 89.9)	0.286
ANB (°)	− 2.4 ± 1.4 (− 5.4 ~ − 0.1)	6.5 ± 2.3 (3.2 ~ 11.5)	< 0.001
Me deviation (mm)	8.7 ± 4.1 (3.3 ~ 17.5)	6.9 ± 3.4 (3.1 ~ 16.4)	0.051
Chin height (mm)	19.3 ± 3.9 (11.6 ~ 26.1)	18.4 ± 3.8 (9.8 ~ 26.5)	0.339
MPA (°)	38.0 ± 5.5 (26.0 ~ 54.8)	38.0 ± 7.9 (17.8 ~ 59.1)	0.997

Comparison of the groups was tested with the Mann–Whitney *U* test except for the gender variable that was tested by the chi-square test

*SD* Standard deviation, *MPA* Mandibular plane angle

### Intra- and inter-group difference in the linear measurements

The differences between the left and right hemimandibular linear measurements in both groups were compared. The results demonstrated that the deviated sides showed narrower condylar widths in both groups. There was no statistically significant difference between the condylar width of the deviated (short) side in group A and the contralateral (long) side in group B. The condylar height, ramus and body length of the contralateral side were longer than those of the deviated side in both groups. However, there was a significant bilateral ramus-proper height difference in group B but not in group A. Ramus-proper height did not show inter-group differences. The condylar width and height, ramus-proper height, and ramus length of the deviated side in group A were similar to those of the contralateral side in group B.

Spatial orientation of the condyle relative to the reference plane did not show bilateral differences in group A. In group B, Con_AP and Con_transv also did not show bilateral differences. However, inter-group difference showed that the right and left condylar heads were positioned more posteriorly, inferiorly and medially in group B than in group A. The contralateral side showed a more anterio-inferiorly and medially-located Go than the deviated side in both groups (all *p* < 0.01) (Table 3).

**Table 3:** Intra- and inter-group difference in the linear measurements

Linear measurements (mm)	Group A (asymmetry with prognathism)			Group B (asymmetry with retrognathism)			Inter-group differences^b^			
	Contralateral side 1)	Deviated side 2)	Bilateral difference^a^ 1)-2)	Contralateral side 3)	Deviated side 4)	Bilateral difference^a^ 3)-4)	1)-3)	1)-4)	2)-3)	2)-4)
	Mean ± SD	Mean ± SD	Difference	Mean ± SD	Mean ± SD	Difference	Mean difference			
Condyle width	19.6 ± 3.0	18.3 ± 3.6	1.3 ± 2.2^**^	17.9 ± 2.7	16.8 ± 3.4	1.1 ± 1.9^**^	1.7^*^	2.8^**^	− 0.4	1.5
Condyle height	22.1 ± 4.3	18.7 ± 4.3	3.4 ± 2.8^**^	17.0 ± 2.9	14.8 ± 2.6	2.1 ± 3.1^**^	5.1^**^	7.3^**^	1.7	3.9^**^
Ramus-proper height	39.6 ± 5.2	38.8 ± 5.2	0.7 ± 3.2	40.4 ± 5.6	38.3 ± 6.1	2.1 ± 3.4^**^	− 0.8	1.3	− 1.5	0.5
Ramus length	62.4 ± 5.9	57.9 ± 6.2	4.5 ± 4.2^**^	58.0 ± 6.1	53.7 ± 6.8	4.3 ± 5.1^**^	4.4^**^	8.7^**^	− 0.1	4.2^**^
Body length	90.0 ± 5.8	87.1 ± 5.1	2.8 ± 3.0^**^	84.1 ± 6.1	81.4 ± 6.2	2.7 ± 2.9^**^	5.9^**^	8.6^**^	3.1^*^	5.9^**^
Con_AP	74.7 ± 3.7	74.8 ± 5.1	− 0.1 ± 3.2	78.8 ± 6.1	80.2 ± 5.9	− 1.3 ± 3.8^*^	− 4.2^**^	− 5.5^**^	− 4.0^**^	− 4.8^**^
Con_vert	74.8 ± 3.7	74.9 ± 5.0	− 0.2 ± 3.0	78.8 ± 6.0	79.7 ± 6.1	− 0.8 ± 4.4	− 4.1^**^	− 5.0^**^	− 3.8^**^	− 5.0^**^
Con_transv	50.2 ± 3.9	50.6 ± 3.6	− 0.4 ± 2.4	48.1 ± 2.9	46.8 ± 4.1	1.2 ± 4.1	2,2^*^	3.4^**^	2.5^**^	3.8^**^
Go_AP	69/9 ± 6.2	72.8 ± 6.6	− 2.9 ± 3.0^**^	79.6 ± 7.7	81.9 ± 7.1	− 2.3 ± 3.2^**^	− 9.7^**^	− 12.0^**^	− 6.8^**^	− 9.0^**^
Go_vert	86.8 ± 8.0	83.5 ± 8.3	3.4 ± 3.7^**^	81.1 ± 7.6	77.9 ± 8.0	3.3 ± 3.7^**^	5.9^**^	9.2^**^	2.5	5.8^**^
Go_transv	44.9 ± 4.4	50.4 ± 4.0	− 5.5 ± 4.4^**^	43.1 ± 4.0	45.8 ± 3.2	− 2.8 ± 3.8^**^	1.9	− 0.8	7.4^**^	4.7^**^

*SD* Standard deviation

^*^p < 0.05

^**^p < 0.01

^a^Paired *t* test for bilateral difference

^b^Mann-Whitney *U* test for the inter-group difference

### Intra- and inter-group difference in the angular measurements

The sagittal ramal inclination of the deviated side was significantly larger than that of the contralateral side in both groups, indicating a more upright posterior ramal border on the deviated side. The contralateral side of the mandible exhibited a larger gonial angle in group A compared to the deviated side. However, group B did not show any bilateral differences. The gonial angle for both right and left sides was larger in group A than group B (Table 4).

**Table 4:** Intra- and inter-group difference in the angular measurements

Angular measurements (°)	Group A (asymmetry with prognathism)			Group B (asymmetry with retrognathism)			Inter-group differences^b^			
	Contralateral side 1)	Deviated side 2)	Bilateral difference^a^ 1)-2)	Contralateral side 3)	Deviated side 4)	Bilateral difference^a^ 3)-4)	1)-3)	1)-4)	2)-3)	2)-4)
	Mean ± SD	Mean ± SD	Mean difference	Mean ± SD	Mean ± SD	Mean difference	Mean difference			
Axial condyle rotation (yaw)	93.4 ± 6.2	92.8 ± 5.9	0.7 ± 4.5	96.6 ± 5.6	94.8 ± 5.1	1.8 ± 4.4^*^	− 3,2^*^	− 1.4	− 3.9^**^	− 2.1
Frontal condyle inclination (roll)	96.3 ± 6.5	90.5 ± 6.7	5.9 ± 8.4^**^	95.4 ± 6.3	90.9 ± 9.1	4.6 ± 10.6^*^	0.9	5.5^**^	− 5.0^**^	0.4
Axial ramal rotation (yaw)	88.1 ± 6.7	90.6 ± 6.7	− 2.5 ± 12.7	89.9 ± 8.5	90.2 ± 6.7	− 0.2 ± 14.6	− 1.8	− 2.0	0.7	0.4
Frontal ramal inclination (roll)	87.4 ± 7.8	89.9 ± 4.4	− 2.6 ± 10.2	88.6 ± 7.7	90.5 ± 5.0	− 1.9 ± 11.5	− 1.3	− 3.1^*^	1.3	− 0.6
Sagittal ramal inclination (pitch)	87.2 ± 5.7	89.7 ± 5.3	− 2.4 ± 2.3^**^	92.4 ± 7.0	94.2 ± 7.7	− 1.7 ± 4.1^*^	− 5.2^**^	− 6.9^**^	− 2.8	− 4.5^**^
Gonial angle	130.2 ± 7.1	128.9 ± 6.2	1.3 ± 2.9^*^	124.9 ± 6.3	124.5 ± 6.4	0.4 ± 5.3	5.3^**^	5.7^**^	4.0^**^	4.4^**^

*SD* standard deviation

^*^p < 0.05

^**^p < 0.01

^a^Paired *t* test for bilateral difference

^b^Mann-Whitney *U* test for the inter-group difference

### Correlation between the Me deviation and bilateral differences in angular and linear measurements

In both groups, correlations between the degree of transverse deviation of the chin (Me_deviation) and bilateral differences of the mandibular measurements were analyzed. In Group A, the highest correlation was found in condylar height (*r* = 0.681, *p* < 0.01). In group B, the highest correlation was found in the length of the body of the mandible (*r* = 0.583, *p* < 0.01). An increased amount of transverse chin deviation was significantly correlated with the differences in the lengths of the rami in both groups [group A, *r* = 0.604; group B, *r* = 0.410, both *p* < 0.001]. However, no correlation was found for differences in the height of the ramus-proper in groups A and B (Table 5).

**Table 5:** Correlation between the Me deviation and bilateral differences in the measurements

Correlation with Me deviation	Group A (asymmetry with prognathism)		Group B (asymmetry with retrognathism)	
	*r*	*P*	*r*	*P*
Conylar width	0.136	0.417	0.223	0.192
Condylar height	0.681	< 0.001^**^	0.351	0.036^*^
Ramus-proper height	0.216	0.194	0.308	0.068
Ramus length	0.604	< 0.001^**^	0.410	0.013^*^
Body length	0.574	< 0.001^**^	0.583	< 0.001^*^
Con_AP	− 0.057	0.735	0.005	0.975
Con_vert	− 0.082	0.626	− 0.014	0.934
Con_transv	− 0.071	0.671	0.442	0.007^**^
Go_AP	− 0.121	0.469	− 0.222	0.193
Go_vert	0.536	0.001^**^	0.314	0.063
Go_transv	− 0.620	< 0.001^**^	− 0.273	0.107
Axial condyle rotation (yaw)	0.347	0.033^*^	0.081	0.639
Frontal condyle inclination(roll)	0.346	0.033^*^	0.037	0.831
Axial ramal rotation (yaw)	− 0.012	0.945	− 0.324	0.054
Frontal ramal inclination (roll)	− 0.142	0.395	− 0.229	0.178
Sagittal ramal inclination (pitch)	− 0.054	0.748	− 0.195	0.253
Gonial angle	− 0.354	0.029^*^	0.105	0.542

*r*, Pearson correlation coefficient

^*^p < 0.05

^**^p < 0.01

## Discussion

Structural characteristics of 3D asymmetry in different types of skeletal patterns need to be considered to establish an adequate surgical plan. This study aimed to compare structural asymmetry between patients with mandibular prognathism and retrognathism. Since the CT data from each institute have uniform DICOM file formats [21], the pixel data transmission, transformation and 3D analyses were easily and successfully performed.

The 3D morphology of deviated mandibular prognathism has been frequently reported. Patients with prognathism without asymmetry [4, 5, 22, 23] or normal occlusion [6] served as controls in previous studies. However, differences between the pattern of 3D skeletal asymmetry in prognathism and retrognathism have been seldom reported. According to a previous report [11] on 3D skeletal differences between mandibular retrognathic and prognathic patients with facial asymmetry, both groups showed shorter ramus lengths (condylar head to gonion) on the deviated side (short side) than the contralateral side. Since the result of only six measurements were presented, it was difficult to understand the factors related to asymmetric mandibles in different skeletal patterns.

You et al. [5] and Thiesen et al. [24] investigated the characteristics of facial asymmetry with mandibular prognathism. Both group also reported 3D skeletal features of asymmetry with retrognathism separately, using the same study variables in both consecutive studies [20, 25]. Their studies yielded similar findings to the aforementioned results of other report [11]. However, these studies did not directly compare the different subject groups [5, 20, 25] or had limited number of study variables and subject numbers [24].

In our study, the ramus length was defined as the distance from the most superior point of the condyle to Go. To investigate the potential effect of the condylar head better, we measured the condylar height (Con_cranial to Con_dorsal) and ramal-proper height (Con_dorsal to Go) separately. In patients with skeletal class III asymmetry, the ramus length of the two sides are significantly different [4–6, 24]. Data presented in Table 3 show that asymmetric prognathism also exhibited a pattern similar to that observed in previous studies. The differences in ramal and body lengths in group B showed similar pattern to the other reports [5, 6].

However, it was interesting that ramus-proper height, excluding the condylar height, did not show inter-group differences. At the same time, the deviated side in asymmetric prognathism and the contralateral side in asymmetric retrognathism showed similar condylar width and height, ramus-proper height, and ramus height. Goto and Langenbach [19] reported that the condylar height (Con_cranial to Con_dorsal) of the deviated side in the asymmetric group was similar to the control group, whereas the contralateral side was significantly longer than the control (symmetry) group and deviated side. They suggested that the overgrowth of the condylar process on the contralateral side would result in an asymmetric mandible. Other study group also suggested that 3D asymmetry in the ramus would be attributed to the asymmetric spatial orientation of the Gonion rather than the 3D position of the condyle [24]. Although the chin deviation was significantly correlated with the ramal and condylar lengths in mandibular prognathism and retrognathism, no statistically significant correlation with the ramus proper height was found in both groups in our study (Table 5). This result is supported by those of previous study [19]. Furthermore, the results imply that an over-growth of the condylar process on the contralateral side in prognathism and an under-growth of the short side in retrognathism would be an important factor in the development of facial asymmetry. Data concerning condylar volume would provide more insight in the effect of condylar growth on condylar volume and subsequent mandibular asymmetry.

Our results showed that there were significant body length differences in both groups in a similar pattern, which is in agreement with results from previous studies [4, 22, 23]. Therefore, our result and those of others indicate that the body of the mandible follows a similar growth pattern of ramus length in both retrognathic and prognathic asymmetry.

There were no marked differences in right and left 3D condylar positions in patients with asymmetry with prognathism. It was in agreement with previous data [24, 25]. In asymmetric retrognathism, we could find bilateral difference in condyle position on sagittal direction. However, the bilateral difference was smaller than the inter-group difference. The left and right condyles of asymmetric retrognathic patients were positioned more posteriorly, inferiorly and medially compared to asymmetric prognathic patients. This suggested that chin asymmetry did not readily mean an asymmetric condylar position. Even if there is asymmetry in 3D condylar head position, this position would be more affected by the horizontal position of the mandible (retrognathic or prognathic) rather than the magnitude of mandibular asymmetry within each group. The result of correlation analysis in Table 5 also emphasized that there was no significant correlation between the degree of chin asymmetry and condylar position. Even though the difference in transverse condylar position significantly correlated with the degree of chin deviation in asymmetric retrognathism, the bilateral difference was not very great (1.2 ± 4.1 mm).

At the same time, the magnitude of the asymmetric 3D spatial orientation of the condyle in both groups of our study was remarkably smaller than that of Gonion in both group. As also shown in previous studies [24, 25], the results suggests that asymmetric 3D position of condyle is less severe than asymmetric displacement of the gonion point.

In both groups, the mandibular angle (Go) of the contralateral side was shifted more medially, and the mandibular angle of the deviated side was located more laterally, posteriorly and superiorly. At the same time, asymmetric 3D position of the Go also significantly correlated with mandibular asymmetry in prognathism patients, particularly in the vertical and transverse direction. A larger chin deviation in the horizontal direction seemed to also increase the ramus length on the contralateral side, displacing the mandibular angle downward and causing more posterior vertical asymmetry of the mandible. Thus, the spatial position of Go would be the more influential factor in facial asymmetry rather than the 3D condylar position, and surgical procedures to correct this bony asymmetry at the angle of the mandible may be required to obtain an optimal treatment result especially for deviated mandibular prognathism.

In previous reports [4, 6, 24], the contralateral side of the ramus was shifted medially to the side of chin deviation and was more anteriorly inclined in deviated mandibular prognathism. Our results showed a similar pattern of the sagittal ramal inclination reported in previous studies.

Frontal ramal inclination also showed similar pattern but was not statistically significant because of the large individual variations (Table 4). The ramus was in fact more upright or even a little posteriorly inclined in the retrognathic mandible. The bilateral difference in ramus orientation in prognathic patients was more significant than in retrognathic patients, but the absolute left and right differences were smaller than inter-group differences. Therefore, surgeons should consider the correction of the right-left difference in the frontal ramus inclination according to the individual bases. At the same time, the axial rotation of the ramus should not be the critical concern in the correction of mandibular asymmetry in both groups. These bilateral angular measurements of axial, frontal and lateral ramal inclinations exhibited large individual variations, and the degree of chin deviation was not proportional to the asymmetry of the ramus inclination or condylar axis. Therefore, the results suggest that a surgical correction of ramal asymmetry needs to be performed with individualized surgical planning to optimize posterior mandibular symmetry.

The limitation of this study was the lack of control data on normal occlusion from the two different institutes. Even though the demographic variables were not statistically different between the groups in this study, there can be the potential difference of shape and size of the different ethnic groups. Nevertheless, this study has shown different skeletal characteristics of asymmetry by using a large sample of 3D CT data from mandibular prognathism and retrognathism with asymmetry.

## Conclusions

The result of this study demonstrated that the differences in spatial orientation of condyles could not fully explain the chin asymmetry in both groups. Deviated mandibular prognathism or retrognathism are related to asymmetric growth of the condyles. Some of the regional structures of asymmetric mandibles in prognathism and retrognathism exhibited a characteristic pattern of bilateral differences. Asymmetric mandibular body length was the most significantly correlated with chin asymmetry in retrognathic asymmetry patients whereas asymmetric elongation of condylar process was the most important factor for chin asymmetry in deviated mandibular prognathism. These findings would be helpful for clinicians to understand the difference in asymmetrical pattern of these two different groups and contribute to the comprehensive 3D planning of different type of asymmetry.

## Data Availability

The datasets used and/or analyzed during the current study are available from the corresponding author on reasonable request.

## Abbreviations

3D: Three-dimensional
CT: Computed tomography
CBCT: Cone-beam CT
DICOM: Digital imaging and communications in medicine
